# Smoking-induced HLA-DQA1 overexpression in T cells: a novel pathway in thyroid eye disease development

**DOI:** 10.3389/fimmu.2026.1745010

**Published:** 2026-05-07

**Authors:** Yuhe Su, Jiamin Cao

**Affiliations:** 1 Department of Endocrinology, Xi’an No.3 Hospital, The Affiliated Hospital of Northwest University, Xi’an, Shaanxi, China; 2 Department of Ophthalmology, Third Xiangya Hospital, Central South University, Changsha, China

**Keywords:** HLA-DQA1, immune dysregulation, lymphocytes, smoking, thyroid eye disease

## Abstract

**Background:**

Smoking is an established risk factor for thyroid eye disease (TED), its immunopathological mechanisms remain elusive. This study investigates how smoking exacerbates TED through immune cell dysregulation.

**Methods:**

National Health and Nutrition Examination Survey (NHANES) data (1999-2010; n = 62,160) was used to compare leukocyte profiles between smokers and non-smokers using non-parametric tests and meta-analysis, with validation using retrospective clinical data. TED was characterized by orbital immune infiltration via CIBERSORT in the GEO dataset GSE58331 (27 patients vs. 23 controls). Linear regression linked lymphocyte levels with thyroid hormones with validation using retrospective clinical data. Summary-data-based Mendelian randomization (SMR) analyzed causal relationships between blood-derived gene expression and TED risk. Smoking-induced transcriptomic changes were validated using GSE4806 (T cells from smokers vs non-smokers) and RT-qPCR.

**Results:**

Smokers exhibited decreased leukocytes and neutrophils but elevated lymphocytes (p<0.05). Lymphocyte percentages correlated positively with FT3 and TT3, The database findings were consistent with the clinical data results. Orbital tissues showed increased follicular helper T cells and plasma cells in TED patients. SMR confirmed HLA-DQA1 (β=0.76) and HLA-DQB1 (β=0.34) as causal TED risk factors (p-HEIDI>0.05). Smoking induced 406 upregulated genes (including HLA-DQA1/DQB1) and 31 downregulated genes in T cells. RT-qPCR confirmed that HLA-DQA1 expression was significantly upregulated in leukocytes from TED patients who smoke.

**Conclusion:**

Smoking exacerbates TED by elevating T cells and promoting HLA-DQA1 overexpression in peripheral T cells, provide a mechanistic justification for smoking cessation in TED management.

## 1 Introduction

Thyroid eye disease (TED) is an organ-specific autoimmune disease that is influenced by multiple risk factors, among which smoking is a clear risk factor for its onset. At present, evidence from multiple levels can indicate that smoking plays a driving role in the onset of TED. On the one hand, in epidemiological studies, Graves’ patients who currently smoke are more likely to develop TED compared to non-smoking Graves’ patients. In addition, TED patients who smoke have a worse prognosis (1). On the other hand, the proportion of TED patients who smoke and undergo strabismus surgery is higher than that of non-smoking TED patients, indicating that smoking plays a role in the occurrence and severity of strabismus in patients (2). According to the diagnosis and treatment guidelines for TED in China and Europe, smoking is recommended as a measure to control the progression of TED disease (3, 4). However, despite multiple pieces of evidence confirming the involvement of smoking in the pathogenesis of TED, how this process occurs at the micro level remains to be explored.

Current research has shown that smoking can cause changes in signaling pathways within the body. Specifically, smoking can directly lead to an increase in cellular oxidative stress levels. For example, treating orbital fibroblasts with cigarette extracts can induce inflammatory phenotype transformation and increase intracellular oxidative stress levels, activating the RAGE signaling pathway (5). On the other hand, smoking can also be associated with a variety of other autoimmune diseases, such as type 1 diabetes and inflammatory bowel disease (6–8). The common important feature of these autoimmune diseases is the disorder of the immune system in the body, which suggests that smoking may be involved in the regulation of the immune system. Existing research also suggests that smoking can cause pathological changes in peripheral blood white blood cells, such as pro-inflammatory changes in monocytes (9). However, it is still unclear whether smoking can affect the onset and progression of TED disease through changes in peripheral blood white blood cells.

This study aims to explore the hypothesis that smoking causes TED disease through changes in peripheral blood white blood cells. Using public data for epidemiological, causal, and gene chip analyses, we will clarify the impact of smoking on the immune system during TED disease and expand our understanding of the pathogenesis of TED.

## 2 Methods

### 2.1 NHANES database

To obtain clinical data of the population, this study used the NHANES database (https://wwwn.cdc.gov/nchs/nhanes/Default.aspx). Download the data and obtain six datasets: 1999-2000, 2001-2002, 2003-2004, 2005-2006, 2007-2008, and 2009-2010. Obtain demographic data, smoking data, and peripheral blood data from six datasets separately; Obtain thyroid function data of the population from the 2007–2008 and 2009–2010 datasets. Among them, demographic data was obtained from Demographics Data, and the total number of people included in the study was determined based on the Respondent sequence number. The smoking data is sourced from Questionnaire Data and obtained from the population through SMQ620- Ever trimmed cigarette smoking in Smoking Cigarette Use. The peripheral blood data is sourced from Laboratory Data and obtained through Complete Blood Count with 5-part Differential Whole Blood LBXWBCSI - White blood cell count (1000 cells/uL), LBXLYPCT - Lymphocyte percent (%), LBXMOPCT - Monocyte percent (%), LBXNEPCT - Segmented neutrophils percent (%), LBXEOPCT - Eosinophils percent (%), LBXBAPCT - Basophils percent (%). Obtain peripheral blood data. Thyroid function data is sourced from Laboratory Data and obtained through the Thyroid Profile LBXT3F - Triiodothyronine (T3), free (pg/mL), LBXT4F - Thyroxine, free (ng/dL), LBXTGN - Thyroglobulin (ng/mL), LBXTSH1 - Thyroid stimulating hormone (uIU/mL), LBXTT3 - Triiodothyronine (T3), total (ng/dL), LBXTT4 - Thyroxine, total (T4) (ug/dL) Obtain thyroid data. All significance analyses were set to P<0.05.

### 2.2 NHANES source data processing

In order to integrate data from different sources in NHANES, this study merged the data using Respondent sequence numbers. In this study, NHANES data analysis was divided into two parts. The first part is to clarify the relationship between smoking and peripheral blood and its components. In this study, data preprocessing was first performed, retaining only values of SMQ620 = 1 or 2. Afterwards, the Shapiro-Wilk test was conducted to determine whether the data of peripheral blood and its components had normality. Group the data according to the SMQ values, compare the peripheral blood and component values of the two groups of people in six datasets using the Mann-Whitney U test, and merge the effect values of the six datasets using the Random Effects model in meta-analysis. Calculate and plot using the R language (4.4.3).

The second part is to clarify the relationship between lymphocytes and thyroid function. Because only the 2007–2008 and 2009–2010 datasets contain Thyroid Profile data, the data from 2007–2008 and 2009–2010 were merged, and the relationship between lymphocyte percentage and FT3, FT4, TT3, TT4, TSH, and Tg was clarified through simple linear regression analysis. Analyze and plot data using GraphPad 10.

### 2.3 GEO data analysis

To investigate the infiltration of lymphocytes and gene expression in tissues, this study used the Gene Expression Omnibus database (GEO database) (https://www.ncbi.nlm.nih.gov/geo/) to download the corresponding gene chip data and calibrate the data. This study is divided into two parts. The first part downloads GSE58331 and GSE231693, and analyzes the infiltration of immune cells in tissues using the CIBERSORT algorithm (10). The second part is to clarify the genetic changes in T lymphocytes and B lymphocytes caused by smoking. Download GSE4806 and GSE18723 chip data from the GEO database to clarify the transcriptional changes in T lymphocytes and B lymphocytes caused by smoking. Compare smoking and non-smoking samples using the GEO2R tool in the GEO database to identify changes in gene transcription. Set a P-value less than 0.05 and an absolute value of Log2 (Fold Change) greater than 1 as differentially expressed genes, and use differentially expressed genes for Kyoto Encyclopedia of Genes and Genomes (KEGG) and Gene Ontology (GO) analysis to clarify the signaling pathways of differential gene enrichment Biological Process(BP)、Cellular Component(CC)、Molecular Function(MF)。 Perform KEGG and GO analysis and plot using the R language.

### 2.4 SMR

Using SMR of expression quantitative trait locus (eQTL) (https://yanglab.westlake.edu.cn/software/smr/#Overview), explore the causal relationship between gene expression in blood and TED. Among them, the GWAS data for gene expression in blood was derived from the V8 release of the GTEx eQTL/sQTL summary data, and the whole blood eQTL dataset was selected, which includes 670 donors. TED’s GWAS summary data is sourced from the GWAS database in Finland (https://www.finngen.fi/en/access_results). It contains 412181 samples, including 3176 TED patients and 409005 control group. Use the 1000 Genomes European reference to remove linkage disequilibrium. SMR analysis was conducted using Windows PowerShell, and heterogeneity was tested using the Heterogeneity in Dependent Instruments (HEIDI) test. p-HEIDI values greater than 0.05 were considered non-pleiotropy. Set false discovery rate (FDR)<0.05 to indicate genes with causal relationships. Visualize the results using the R language.

### 2.5 Clinical data validation

To validate the relationship between smoking and leukocytes, clinical data from patients diagnosed with TED between 2018 and 2023 were retrieved from the Central South University Xiangya Third Hospital case management system. The dataset included patient sex, age, smoking history, clinical activity score, intraocular pressure, corticosteroid use (within 3 months), ocular surgery (within 3 months), and complete blood count results. Patients were classified as non-smokers based on self-report of never having smoked before disease onset. Based on their smoking history, patients were categorized into smoking and non-smoking groups. Age, thyroid function parameters, and leukocyte subset counts are presented as mean ± standard deviation (SD). Clinical activity score and intraocular pressure are presented as median (first quartile, third quartile). An independent samples t-test was employed to compare the following parameters between the two groups: total white blood cell count, and the percentages of monocytes, lymphocytes, granulocytes, eosinophils, basophils and clinical activity score in peripheral blood.

To further investigate the correlation between lymphocytes and thyroid function, lymphocyte and thyroid function test data from 2018 to 2023 were collected from the same system, without restrictions on patient sex, age, or disease status. Simple linear regression analysis was conducted to assess the relationships between lymphocyte levels and FT3, FT4, TT3, TT4, TSH, and Tg. A p-value of less than 0.05 was considered statistically significant.

### 2.6 Reverse transcription quantitative PCR

To compare the expression levels of specific genes in peripheral blood leukocytes between TED patients and healthy controls, peripheral blood samples (2 ml each) were collected from 3 TED patients who smoked and 3 non-smoking healthy controls. Following collection, a 10x red blood cell lysis buffer was added to the whole blood. After 10 minutes of incubation, the mixture was centrifuged at 2000 rpm to obtain the leukocyte pellet, which was subsequently washed twice with PBS buffer. Total RNA was extracted from the leukocytes using 1 ml of TRnaZol Reagent (New Cell & Molecular, China). After a 5-minute incubation, an RNA extraction buffer was added to complete the isolation. The resulting RNA template was reverse-transcribed into cDNA using a commercial kit (GenStar, Kangrun Biotechnology).

The expression levels of HLA-DQA1 and HLA-DQB1 in the cDNA from both the TED and control groups were quantified by qPCR and compared using a t-test. The primer sequences used in this study are listed below:

GAPDH-F: 5’-GGAGCGAGATCCCTCCAAAAT-3’
GAPDH-R: 5’-GGCTGTTGTCATACTTCTCATGG-3’
HLA-DQA1-F: 5’-TCGCTCTGACCACCGTGAT-3’
HLA-DQA1-R: 5’-AGGGACCGTAAAACTGGTACAA-3’
HLA-DQB1-F: 5’-ACCTTCGGGTAGCAACTGTC-3’
HLA-DQB1-R: 5’-AAATCCTCGGGAGAGTCTCTG-3’

## 3 Result

### 3.1 Smoking can cause changes in the peripheral blood white blood cells

**Figure 1:**
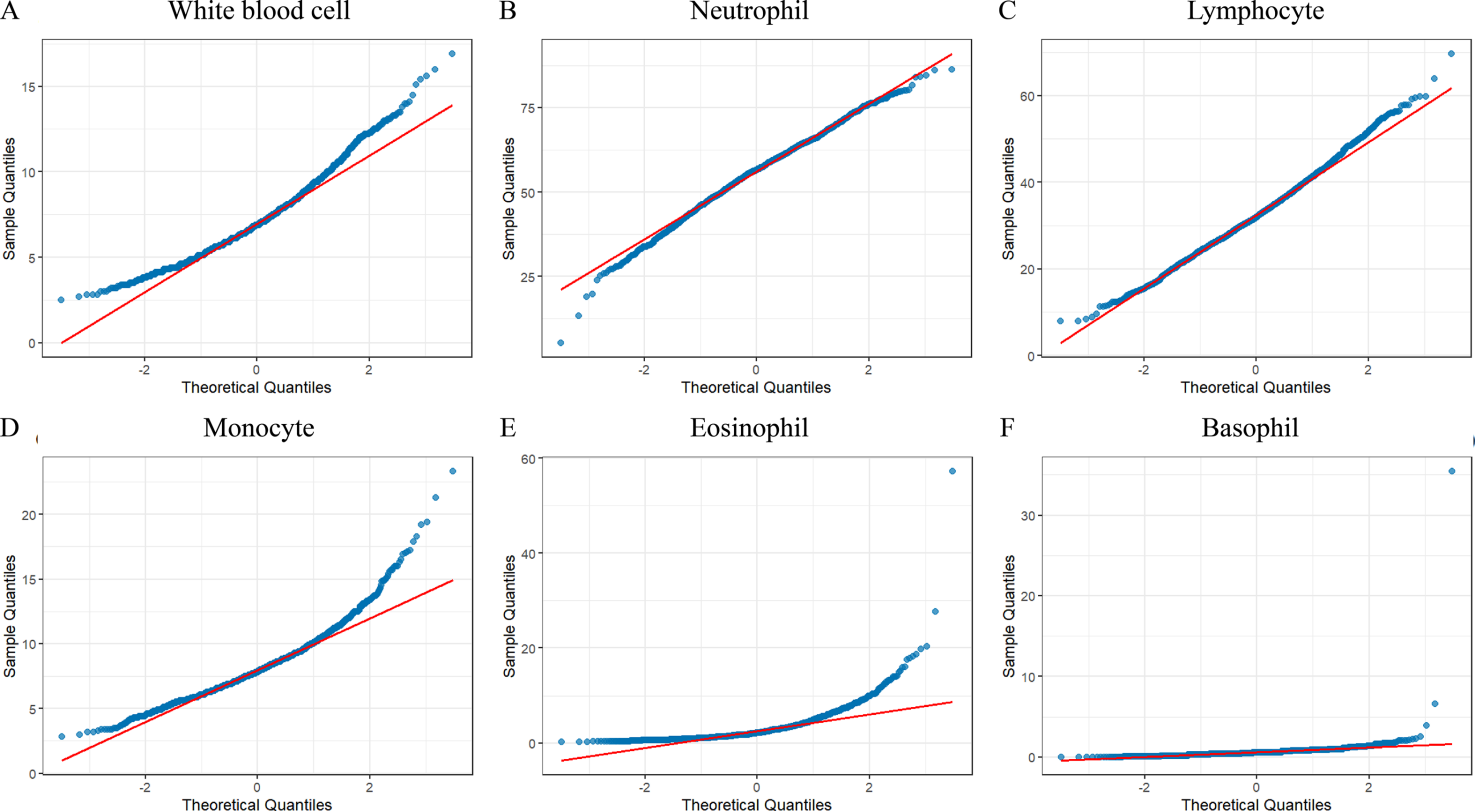
Normality test: Test the normality of total white blood cell count **(A)**, neutrophils **(B)**, lymphocytes **(C)**, monocytes **(D)**, eosinophils **(E)**, and basophils **(F)**.

**Figure 2:**
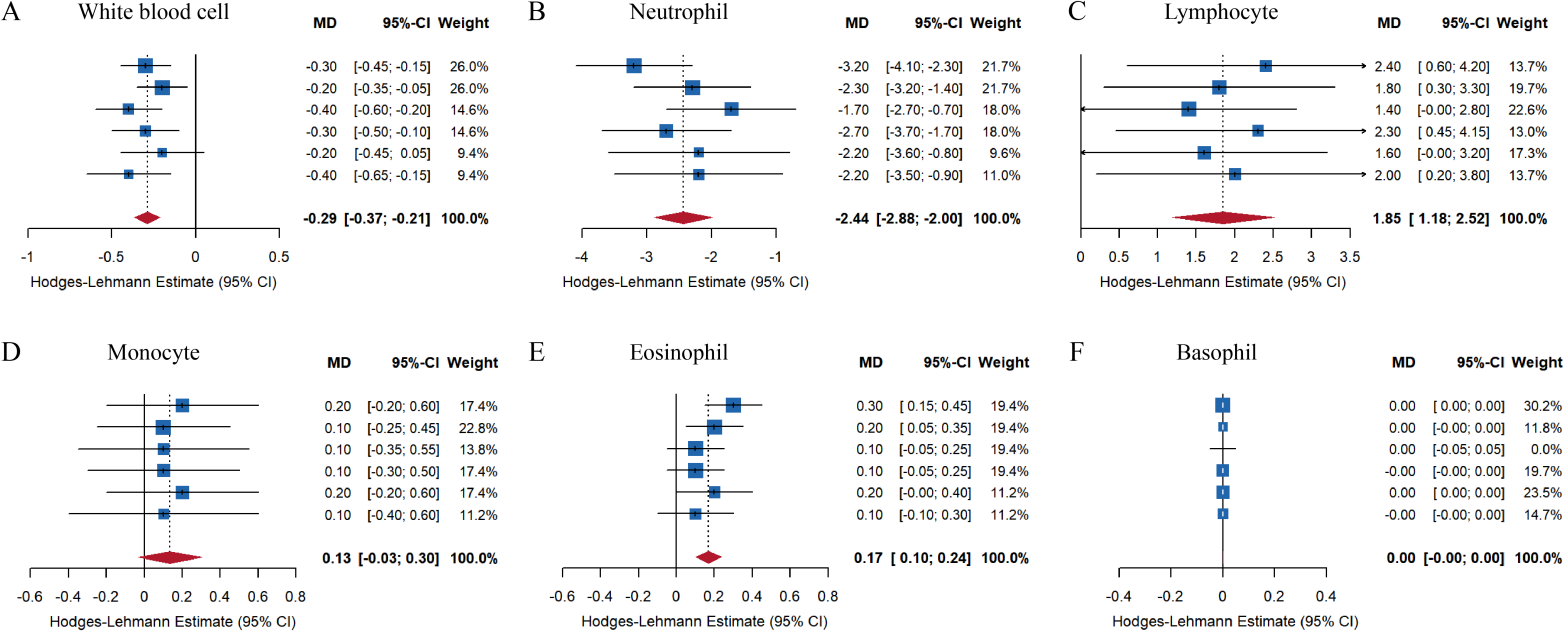
Impact of smoking on white blood cells and their components. The population was grouped based on whether they had smoked in the past, and the values of total white blood cell count **(A)**, neutrophils **(B)**, lymphocytes **(C)**, monocytes **(D)**, eosinophils **(E)**, and basophils **(F)** were compared between the two groups. The total effect values of 12 years of data were combined.

The data on smoking and peripheral blood from 1999 to 2010 were obtained through the NHANES database, with a total of 62160 cases included in the population data. Among them, the number of people with SMQ = 1 or 2 was 10737. Firstly, normality testing was performed on the data, and the results showed that the values of various components in the peripheral blood of the population, including total white blood cells, neutrophils, lymphocytes, monocytes, eosinophils, and basophils, exhibited skewed distribution (**Figure 1**). Then, grouping was conducted based on whether the population smoked, and the relationship between smoking and white blood cells was explored. Non-parametric rank sum tests were used to test the white blood cell values of two groups of participants in six datasets, and meta-analysis was conducted to merge the total effect values of the six datasets. The results showed that compared to the non-smoking population in the past, the total white blood cell count and neutrophil values were significantly down-regulated in the smoking population (**Figures 2A, B**), while the values of lymphocytes and eosinophils were significantly up-regulated (**Figures 2C, E**). There was no significant difference in the values of monocytes and basophils between the two groups (**Figures 2D, F**).

### 3.2 Retrospective clinical study validates the impact of smoking on peripheral blood leukocytes

To further validate the aforementioned findings, we conducted a retrospective analysis using clinical data extracted from the hospital’s record system. A total of 176 TED patients were included in this analysis, comprising 128 non-smokers and 48 smokers. Detailed clinical characteristics of the cohort are presented in **Table 1**.

**Table 1:** Clinical characteristics of TED patients.

Characteristic	Non-smoker	Smoker
Number	128	48
Age	55.15 ± 14.31	45.44 ± 12.15
Gender	42:86 (male: female)	42:6 (male: female)
Clinical activity score	1 (1, 3)	3 (1.75, 4)
Intraocular pressure	20 (17, 24)	20 (18, 23)
Corticosteroid use (within 3 months)	15:113 (yes: no)	18:30 (yes: no)
Ocular surgery (within 3 months)	4:124 (yes: no)	8:40 (yes: no)
White blood cell (10^9/L)	8.36 ± 4.12	6.81 ± 2.18
Monocyte percentage (%)	5.71 ± 2.12	6.55 ± 1.85
Lymphocyte percentage (%)	22.40 ± 10.48	28.86 ± 8.47
Neutrophil percentage (%)	70.32 ± 11.53	61.58 ± 9.10
Eosinophil percentage (%)	1.13 ± 1.22	2.45 ± 2.11
Basophil percentage (%)	0.40 ± 0.29	0.51 ± 0.28

**Figure 3:**
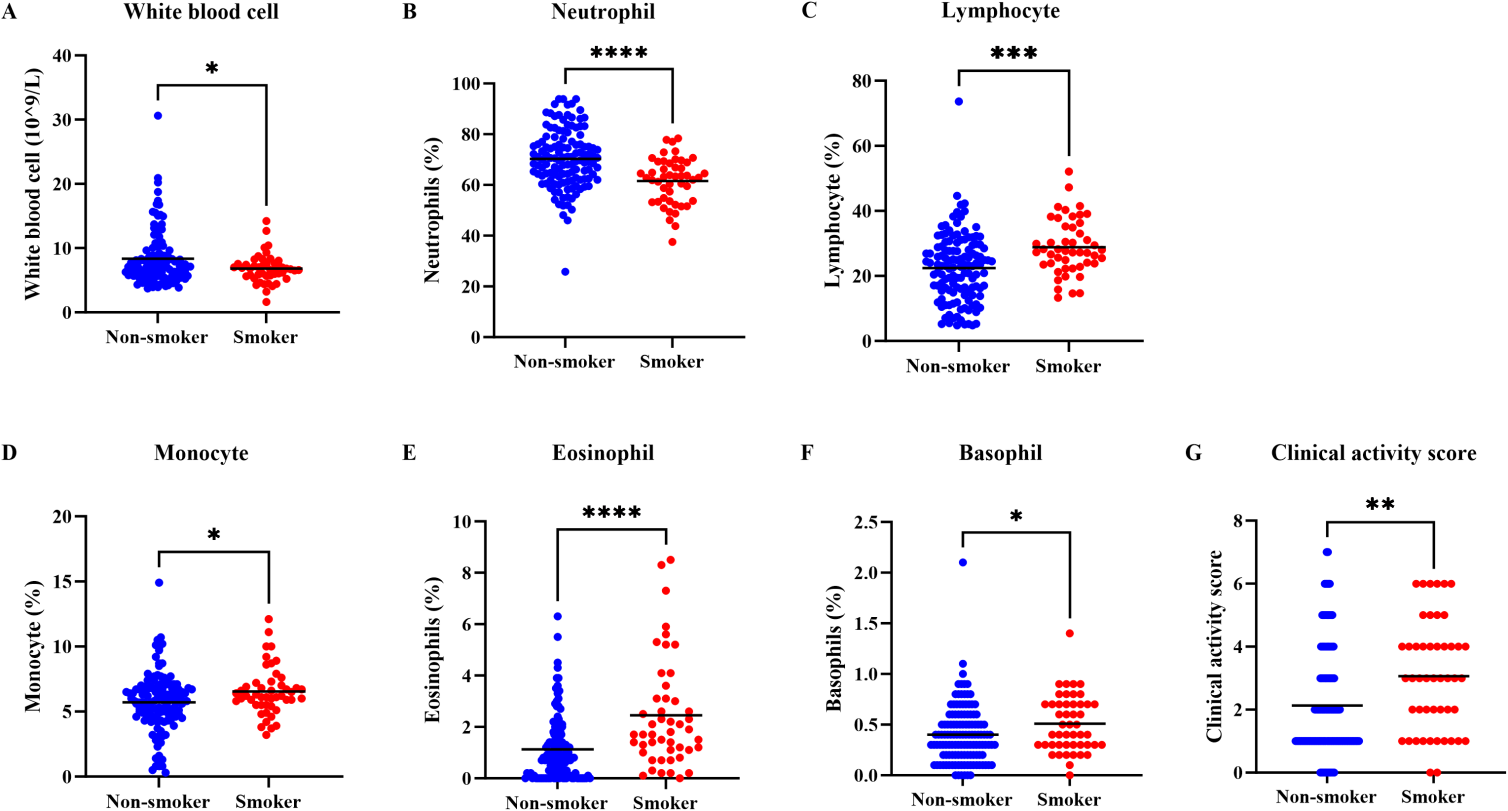
Clinical validation of the effects of smoking on white blood cells and their subsets in TED patients. TED patients were stratified into smoking and non-smoking groups. Comparisons were made between the two groups regarding **(A)** total white blood cell count and the relative percentages of **(B)** neutrophil, **(C)** lymphocyte, **(D)** monocyte, **(E)** eosinophil, and **(F)** basophiland. Comparisons were made between the two groups regarding **(G)** clinical activity score. *P<0.05, **P<0.01, ***P<0.001, ****P<0.0001.

Comparative analysis of leukocyte counts and subset proportions between the two groups revealed that smoking TED patients exhibited a decrease in both the total white blood cell count (**Figure 3A**) and the neutrophil percentage (**Figure 3B**). In contrast, the lymphocyte percentage was significantly higher in smoking patients (28.86% ± 8.47%) compared to non-smoking patients (22.40% ± 10.48%) (**Figure 3C**). Increases were also observed in the percentages of monocytes, eosinophils, and basophils among smokers (**Figures 3D–F**). Comparing the clinical activity score, it was found that TED smokers had a significantly higher clinical activity score than TED non-smokers (**Figure 3G**).

### 3.3 Atlas of immune cell changes in orbital tissue

**Figure 4:**
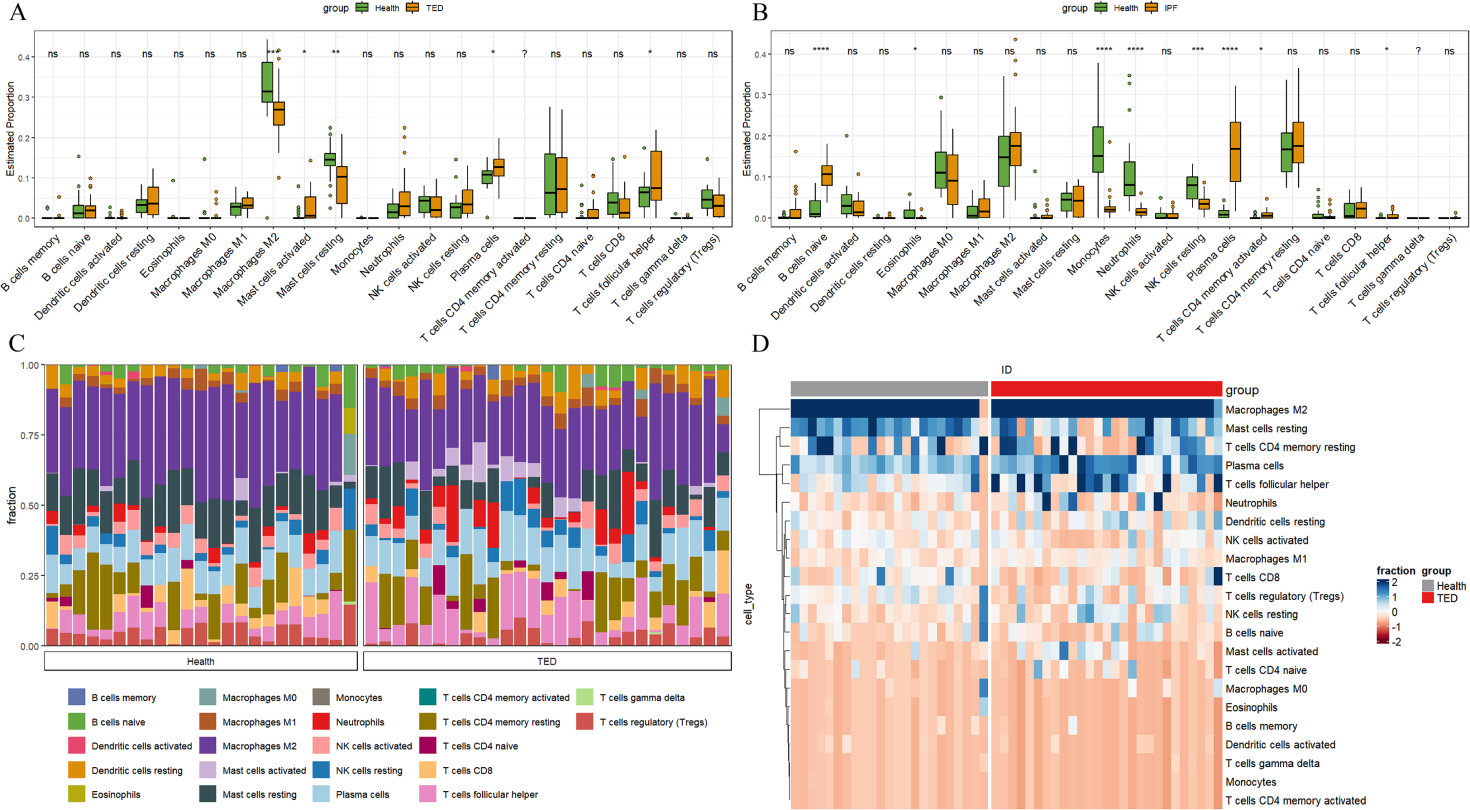
Immunocyte atlas in TED orbital tissues. The immunocyte atlas in TED orbital tissues was explored using the CIBERSORT algorithm **(A)** and validated by intersecting the data from the idiopathic pulmonary fibrosis chip with the TED data **(B)**. The results of the CIBERSORT algorithm were visualized using bar charts **(C)** and heatmaps **(D)**. *: P<0.05, **: P<0.01, ***: P<0.001, ****: P<0.0001.

The previous section clarified that smoking can cause changes in various components of white blood cells in peripheral blood, and it needs to be clarified which immune cells have a more significant impact on TED patients. Therefore, this section downloaded GSE58331 gene chip data, including 23 cases in the normal group and 27 cases in the TED group. The CIBERSORT algorithm was used to determine that the proportion of M2 macrophages and quiescent mast cells (a subtype of eosinophils) in the orbit of TED patients decreased, while the proportion of activated mast cells increased. The proportion of plasma cells and follicular helper T cells was upregulated (**Figure 4A**). Considering that a large portion of cigarette extract enters the circulatory system through the lungs and reaches the eye socket, this study also downloaded chip data on idiopathic pulmonary fibrosis to verify the TED analysis results. The results showed that the proportion of plasma cells and follicular helper T cells was also upregulated in the samples of idiopathic pulmonary fibrosis (**Figure 4B**). Then, the relationship between the proportions of each component cell in the normal group and TED group was characterized by a bar graph (**Figure 4C**), and the values of each sample were characterized by a heat map (**Figure 4D**) to observe the levels of 22 immune cells in TED orbital tissue.

### 3.4 There is a correlation between thyroid function and white blood cells

**Figure 5:**
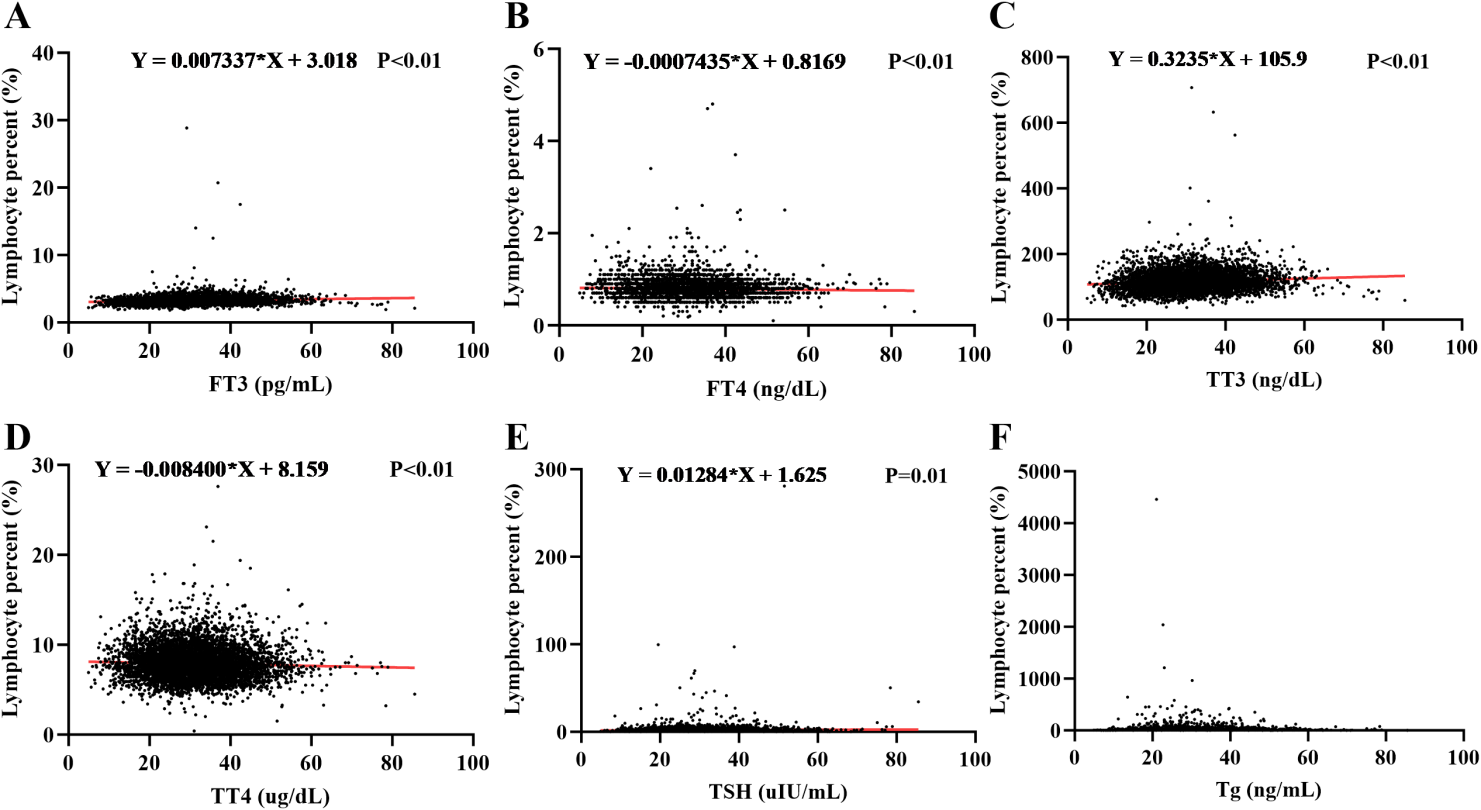
There is a correlation between thyroid function and white blood cells. The relationship between lymphocyte proportion and thyroid function, including FT3 **(A)**, FT4 **(B)**, TT3 **(C)**, TT4 **(D)**, TSH **(E)**, and Tg **(F)**, was clarified through linear correlation analysis.

The previous research has clarified that smoking-induced lymphocyte elevation in the past may be involved in the pathogenesis of TED. To further validate this mechanism. This study used data from NHANES between 2007 and 2010 to construct the relationship between thyroid function and lymphocytes through simple linear regression analysis. The results showed a significant correlation between the increased proportion of lymphocytes and thyroid function. Specifically, the proportion of lymphocytes showed a positive correlation with the levels of FT3, TT3, and TSH (**Figures 5A, C, E**), while showing a negative correlation with FT4 and TT4 (**Figures 5B, D**). The proportion of lymphocytes showed no significant correlation with Tg (**Figure 5F**).

### 3.5 Retrospective clinical study validates the relationship between thyroid function and lymphocytes

To further elucidate the findings described above, a retrospective analysis was conducted using clinically collected data. A total of 2,644 patients were included in this analysis, comprising 813 males and 1,831 females. Detailed clinical characteristics are presented in **Table 2**.

**Table 2:** Clinical characteristics of patients included in the retrospective study on the relationship between lymphocytes and thyroid function.

Characteristic	Value
Number	2644
Age	47.83 ± 15.46
Gender	813:1831 (male: female)
Lymphocyte percentage (%)	27.04 ± 12.81
Free Triiodothyronine (pmol/L)	8.87 ± 8.46
Free Thyroxine (pmol/L)	26.82 ± 18.24
Triiodothyronine hormone (nmol/L)	2.58 ± 1.50
Thyroxine hormone (nmol/L)	146.88 ± 59.25
Thyroid Stimulating Hormone (μIU/ml)	1.94 ± 5.72
Thyroglobulin (ng/ml)	56.41 ± 98.81

**Figure 6:**
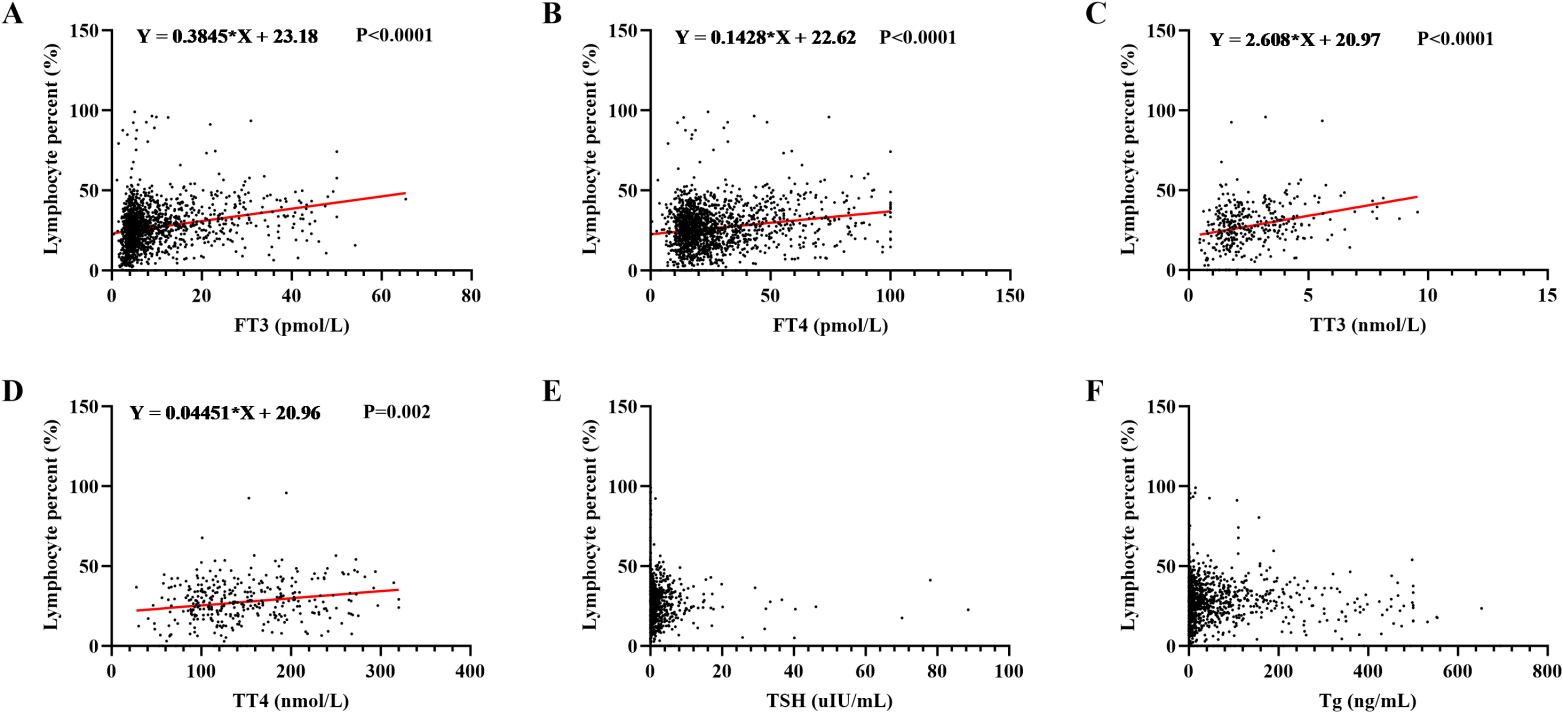
Clinical validation of the correlation between thyroid function and lymphocyte percentage. Retrospective clinical data were analyzed to assess the correlations between lymphocyte percentage and levels of **(A)** FT3, **(B)** FT4, **(C)** TT3, **(D)** TT4, **(E)** TSH, and **(F)** Tg.

Simple linear correlation analyses were performed to assess the relationships between the lymphocyte percentage and levels of FT3, FT4, TT3, TT4, TSH, and Tg. The results revealed significant positive correlations between the lymphocyte percentage and both FT3 and TT3, which is consistent with the NHANES findings (**Figures 6A, C**). Whereas, significant positive correlations were also observed between the lymphocyte percentage and both FT4 and TT4, which differed from the NHANES results (**Figures 6B, D**). No significant correlations were found between the lymphocyte percentage and TSH or Tg (**Figures 6E, F**).

### 3.6 SMR analysis of TED onset

**Figure 7:**
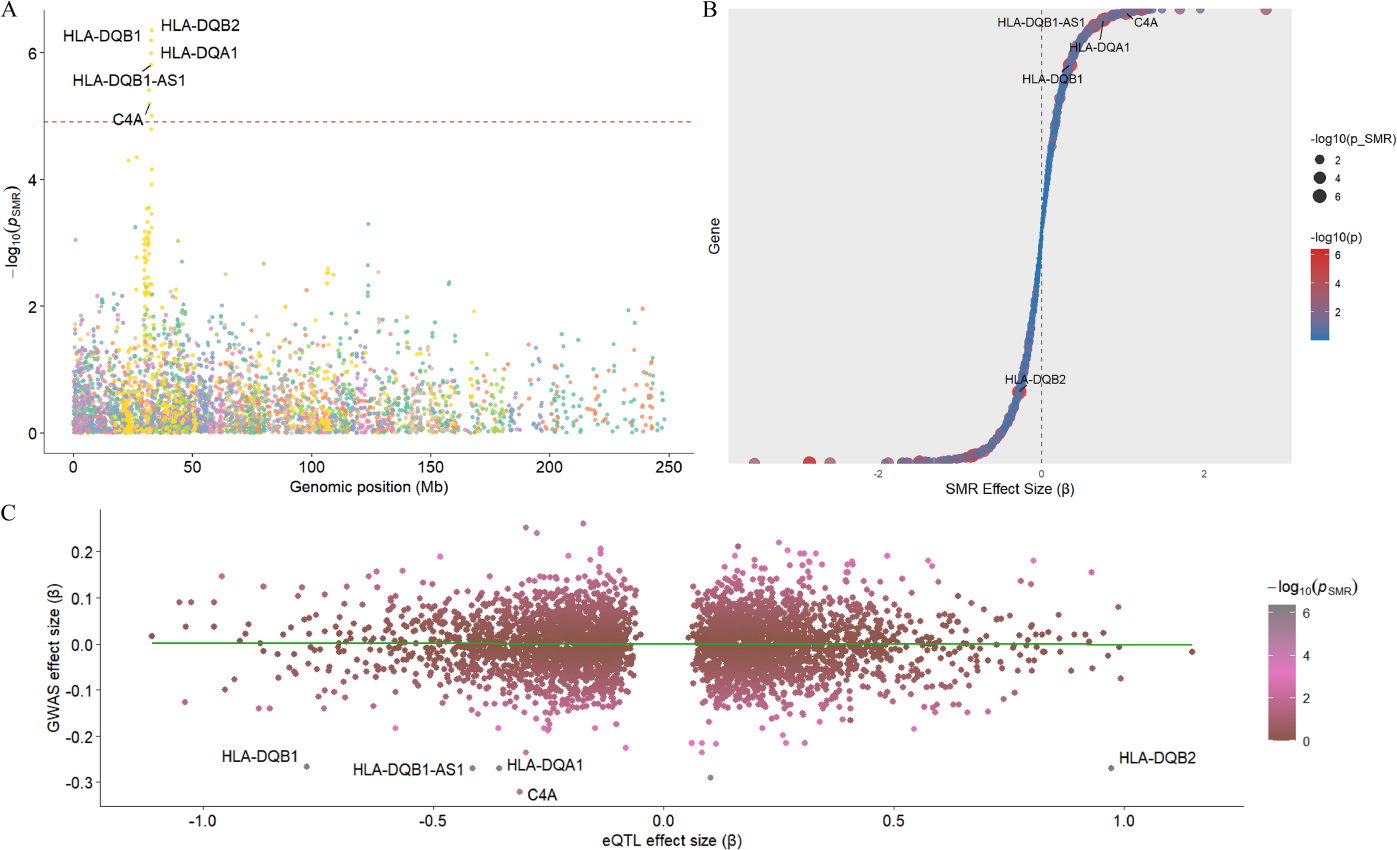
SMR analysis of TED pathogenesis. Through SMR analysis, five genes were identified as having a causal relationship with the pathogenesis of GO, including C4A, HLA-DQA1, HLA-DQB1, HLA-DQB1-AS1, and HLA-DQB2 **(A–C)**.

In order to further clarify which genes in lymphocytes have a causal relationship with the onset of TED, this study used GWAS data from blood tissue and TED, with blood tissue data as exposure factors and TED data as outcomes. After SMR analysis, a total of 5 genes with causal relationships with TED were obtained, including C4A, HLA-DQA1, HLA-DQB1, HLA-DQB1-AS1, and HLA-DQB2 (**Figure 7A**). Among the 5 genes, C4A(b = 1.03)、HLA-DQA1(b = 0.76)、HLA-DQB1(b = 0.34)、HLA-DQB1-AS1(b = 0.65) is a risk factor for TED, while HLA-DQB2 (b=-0.27) is a protective factor for TED (**Figures 7B, C**). In terms of pleiotropy testing, the HEIDI values of all five genes are greater than 0.05.

### 3.7 Smoking can cause upregulation of HLA-DQA1 and HLA-DQB1 in T lymphocytes

**Figure 8:**
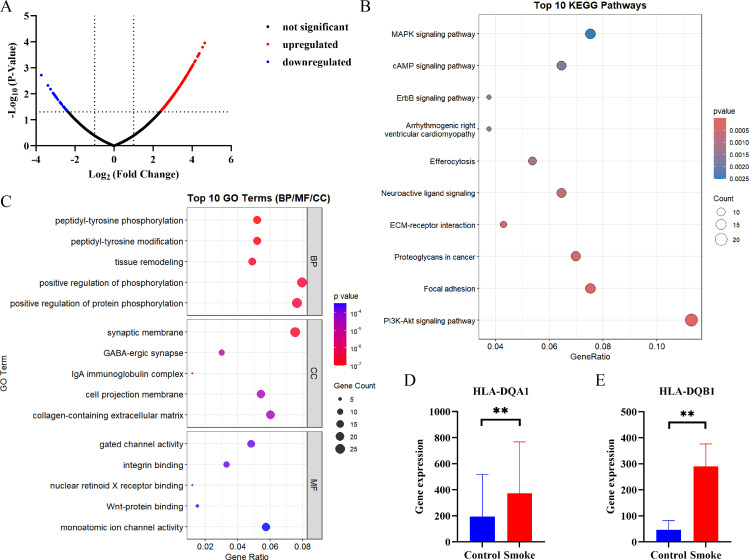
Smoking can induce upregulation of HLA-DQA1 and HLA-DQB1 in lymphocytes. GSE4806 gene chip analysis identified changes in the transcriptome levels of T lymphocytes in smoking patients compared to non-smoking patients **(A)**; differential expression genes were used for KEGG **(B)** and GO **(C)** analysis; T-test confirmed upregulation of HLA-DQA1 **(D)** and HLA-DQB1 **(E)** expression in T lymphocytes of smoking patients, the data are presented as mean ± SD. **P<0.01.

The previous section clarified that five genes in the blood have a causal relationship with the onset of TED. In order to further clarify the changes in the transcriptome of blood cells caused by smoking, this study used GSE4806 and GSE18723 chip data to determine the effects of smoking on T lymphocytes and B lymphocytes, respectively. The GSE4806 chip identified a total of 406 upregulated genes and 31 downregulated genes (**Figure 8A**). 259 pathways were identified through KEGG enrichment analysis, and the top 10 pathways with the lowest P-values are shown in **Figure 8B**. The GO pathway analysis identified pathway changes in the BP, CC, and MF components of T lymphocytes in smoking patients (**Figure 8C**). Finally, the T-test confirmed that the expression levels of HLA-DQA1 and HLA-DQB1 on T lymphocytes were elevated in smoking patients (**Figures 8D, E**). There was no significant difference in the expression levels of HLA-DQA1 and HLA-DQB1 between smoking and non-smoking samples in the GSE18723 chip.

### 3.8 RT-qPCR determines HLA-DQA1 and HLA-DQB1 expression in TED

**Figure 9:**
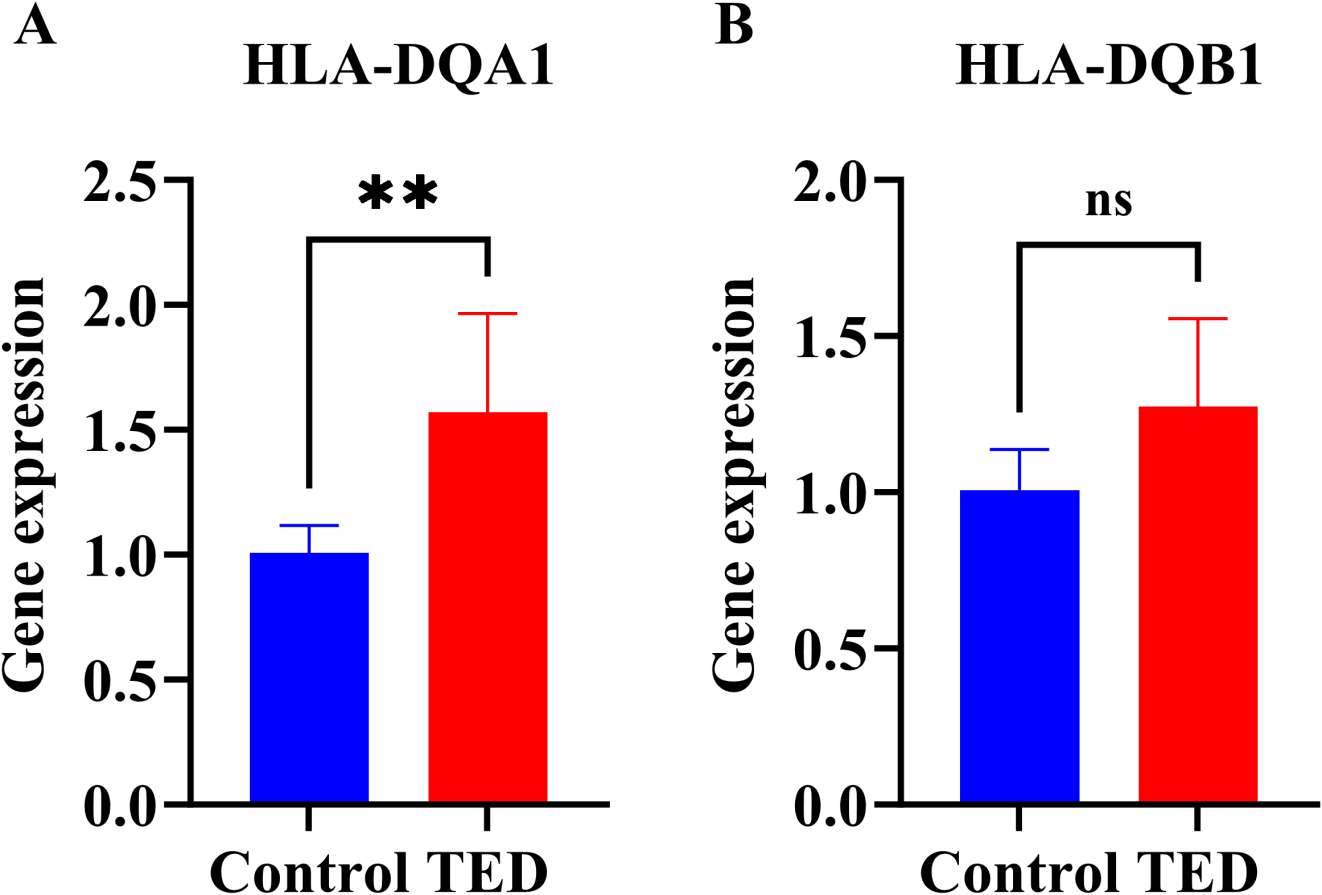
Detection of HLA-DQA1 and HLA-DQB1 expression. RT-qPCR analysis of **(A)** HLA-DQA1 and **(B)** HLA-DQB1 expression levels in peripheral blood leukocytes from non-smoking healthy controls and smoking TED patients. The data are presented as mean ± SD. **P<0.01.

To further elucidate the expression profiles of HLA-DQA1 and HLA-DQB1 in TED patients relative to healthy controls, we conducted an *in vitro* RT-qPCR assay using peripheral blood samples from 3 TED patients who smoked and 3 non-smoking healthy controls. The results demonstrated that the expression level of HLA-DQA1 was significantly higher in the smoking TED group compared to the non-smoking controls (P < 0.05) (**Figure 9A**). In contrast, no significant difference in HLA-DQB1 expression was observed between the two groups (**Figure 9B**).

## 4 Discussion

In this study, the relationship between smoking and peripheral blood was first clarified through the NHANES database. Through retrospective evidence over 12 years, it was found that smoking can cause changes in various white blood cells in the body, which provides a basis for exploring the impact of smoking on TED through immune cells. In order to further clarify which type of white blood cell is causing the progression of the disease, this study used chip data from the GEO database and identified infiltrating white blood cells in tissues and elevated levels of lymphocytes through the CIBERSORT algorithm. This result directly led to subsequent research on whether the elevation of these lymphocytes is related to the pathogenesis of TED. To investigate this issue, this study further clarified the relationship between lymphocyte expression levels and thyroid function, revealing a positive correlation between the two. Furthermore, in order to further clarify the causal relationship between protein expression in blood and TED, this study used SMR analysis to identify the causal relationship between the expression of five proteins and diseases. To verify this result, this study once again used chip data from GEO to clarify that smoking can cause an increase in the expression levels of HLA-DQA1 and HLA-DQB1 in lymphocytes.

The pathogenesis of autoimmune diseases is complex, with both similarities and differences among different autoimmune diseases. Overall, the onset of such diseases is characterized by immune disorders, where the immune system in the body incorrectly recognizes the antigens it expresses and activates the immune process, causing tissue damage (11). For TED, currently known pathogenic self-antigens include thyroid-stimulating hormone receptors and insulin-like growth factor 1 receptors (12). When exposed to external stimuli, including excessive and ectopic expression of thyroid stimulating hormone receptors in the body (using adenovirus loaded with TSHR - α subunit to construct TED mouse models), thyroidectomy surgery, radiation therapy, etc., antibodies targeting TSHR can be produced in the body, further causing TED disease (13). In addition, evidence from epigenetic studies also suggests that changes in the *in vivo* and *in vitro* environment can cause epigenetic disorders in TED patients, including abnormal expression of non-coding RNAs (14). However, the onset of TED not only includes external stimuli but also genetic risk factors. For example, in recent years, the polymorphism of multiple genes has been considered to be associated with the pathogenesis of TED, and the differences in gene expression levels may be involved in the pathogenesis of TED. This study mainly explores the mechanism of the impact of smoking on the pathogenesis of TED.

Smoking was explicitly involved in the pathogenesis of TED a long time ago. Most of these studies are epidemiological studies, but the micro-regulatory mechanisms underlying the incidence of TED caused by smoking are not clear. By comparing the role of smoking in other autoimmune diseases horizontally, existing research suggests that smoking can alter the normal phenotype of pathogenic cells by promoting oxidative stress levels. For example, smoking can increase the level of oxidative stress in type I diabetes mice (15). Tobacco extract can also cause changes in the gut microbiota of mice with inflammatory bowel disease (16). In this study, to further narrow down and clarify the immune cell changes caused by smoking, gene chips from patients with idiopathic pulmonary fibrosis were used to analyze and intersect with differential levels of immune cells at TED. The reason for this choice is twofold: on one hand, it is due to the clear etiology of smoking induced idiopathic pulmonary fibrosis; on the other hand, idiopathic pulmonary fibrosis also has the pathogenesis of autoimmune disorders and fibrotic changes, and the pathogenic substances of smoking usually enter the circulation through the lungs and reach the eye sockets.

Smoking can cause pathological changes in multiple systems within the body. Smoking-induced dysregulation of T cell subset ratios plays a pivotal role in disease initiation and progression (17). When Treg cells are downregulated, the regulatory mechanism that maintains autoimmune tolerance is impaired, and downstream immune regulation activated by autoantibodies is imbalanced, leading to disease onset (18). For example, smoking can promote neutrophil NETs through IL-35 and activate the STAT3 pathway, recruit dendritic cells, and cause downregulation of the Treg differentiation ratio (19). In addition, smoking can downregulate the expression of MR1 molecules and block the role of MR1 in mucosa-associated T cells, thereby inhibiting the physiological function of these cells (20). This study clarified that smoking can cause an increase in the proportion of plasma cells and follicular helper T cells in TED patients. Follicular helper T cells in the germinal center can promote the production of humoral immunity, while plasma cells can produce a large amount of antibodies. The upregulation of these two types of cells can cause an increase in autoantibodies in TED cells, thereby activating antibody-induced cytotoxicity (21).

HLA genes are a group of genes related to immune response, which can be roughly divided into Class I and Class 2 gene groups. HLA-1DQA1, as an important component of the human major histocompatibility complex, are considered to be associated with the onset of various diseases, one of which is autoimmune diseases, including endocrine autoimmune diseases (22). In recent years, studies have also reported autoimmune diseases of the digestive system caused by HLA-DQA1, including primary biliary cholangitis (23). In TED diseases, studies have shown that certain alleles in HLA-DQA1 are risk factors for TED (24). However, the roles played by these alleles seem to vary among different ethnic groups. For example, the frequency of HLA-DQA1 * 0201 in the Iranian population is significantly reduced in Graves’ disease (25). This study used the SMR method and European ethnic genetic data to clarify that HLA-DQA1 are risk factors for TED, which is clearer than the causal relationship obtained from case-control studies.

There are three shortcomings in this study. Firstly, to clarify which type of white blood cell promotes the pathogenesis of TED, this study utilized gene chip data. However, in this process, the sample source of chip data was orbital tissue, while the sample source of NHANES data was blood. Further investigation is needed to determine whether immune cells are consistent between the two samples. In addition, the chip data clearly states that follicular helper T cells are elevated cells, but this subtype of T cells cannot replace the overall increase in T cell levels in the blood; Secondly, this study used SMR analysis to clarify the causal relationship between the elevation of HLA-DQA1 in the blood and the onset of TED. However, it is currently unknown whether the elevation of HLA-DQA1 in the blood is entirely caused by the presence of HLA-DQA1 in lymphocytes. Third, due to limitations in experimental conditions, this study only employed RT-qPCR for basic experimental validation and did not perform more direct experimental verification of the findings.

## 5 Conclusion

This study clarified that smoking can affect the pathogenesis of TED by increasing the levels of T lymphocytes in peripheral blood. At the molecular level, the increased expression levels of HLA-DQA1 in lymphocytes caused by smoking are causally related to the elevation of TED.

## Data Availability

The raw data supporting the conclusions of this article will be made available by the authors, without undue reservation.
